# Book Notices

**Published:** 1884-07-20

**Authors:** 


### *BOOK NOTICES.*

***Medical Advice to Young Men***, by C. A. Bryce, M.D., Richmond, Va.

This little work is well-timed and appropriate, being designed to give clear and truthful ideas to the young men of the country who are being victimized by the quacks in relation to "Lost Manhood, Weakness and Decay," "Youthful Indiscretions," etc., etc. The works whicb have been written upon this subject are, as a class, wholly empirical, and designed to excite the fears of the young and ignorant, that the authors may fleece them of their money. The loss of money is, unfortunately, not the only evil that results. Sensitive minds are alarmed, and often driven to despair and insanity, where no such result is necessary. Not so with this little work, which, though small, may be read with interest and profit by any one desiring information on this subject. It does not supplant the family physician, but points out the true dangers incident to youthful indiscretion, how to avoid them and when a physician should be consulted.

The best method of combatting quackery is to educate the masses in the truths of science. This book looks in that direction, and is, therefore, to be commended.

***The Essentials of Anatomy, Physiology and Hygiene***: A Text Book for Schools and Academies, by Roger S. Tracv, M.D., Sanitary Inspector of the New York City Health Department; Author of the Hand Book of Sanitary Information for Householders. New York: D. Appleton & Co., 1, 3 and 5 Bond St. 1884.

This is a school book, and is, in all respects, admirably adapted to the non-professional student, as everything is presented in language plain and intelligible, well illustrated, and suffieiently full to contain all the leading and essential parts and principles of physiological science.

The arrangement of the work, its size and its typographical execution, etc., are all good, and—like everything that emanates from that most excellent and reliable Publishing House, D. Appleton & Co.—it is well gotten up, and deserving of the confidence and patronage of the public.

***Health Hints for Travelers***, by John C. Lundborg, M.D. D. G. Brinton, Philadelphia. 1884.

An interesting and useful little work of sixty-one pages, designed to teach the traveler how to keep well on the various changes and exposures which he must encounter. The Author, having himself been an extensive traveler, is the better qualified for the task by drawing from his own observations and experience.

***Auscultation, Percussion and Urinalysis***, an Epitome of the Physical Signs of the Heart, Lung, Liver, Kidney and Spleen in Health and Disease. Edited by C. Henri Leonard, A.M., M.D., Professor of the Medical and Surgical Diseases of Women and Clinical Gynecology, Michigan College of Medicine, Detroit. Fully illustrated.

This little work is very useful and practical, particularly in the department of Physical Diagnosis. It was designed for the use of students, but may be read with interest and profit by the practitioner.

*First Application of Waring's Sewerage System in Paris*, a Pamphlet published in French by Ernest Pontzen—Reviewed by E. Van Goidtsnoven, M.D.

To those who are familiar with French and interested in the study of the most modern and approved plans of sewerage, the above pamphlet is particularly recommended. Mankind is so constituted that it seldom, if ever, awakes to the importance of averting the countless dangers that threaten its very existence uutil the mowing scythe of death forces it to realize that "Famine and plague are sent as scourges for amendment." We might also say, *for reform*.

The remembrance of the terrible pestilence in Memphis is still fresh in the minds of our readers. Twenty-two fearful incursions of yellow fever within fifty years forced that city to recognize that she was, in a great measure, responsible for her own distress and suffering, and that she had been too long indifferent to the "condition of her bowels." There, as in many old cities, fœcal matters were deposited in vaults dug in the ground, in proximity to habitations. A portion of the matters so introduced filtered away into the soil; the rest was removed when the vault became full. Think of the emanations rising from these cesspools of death under a July sun! Realizing her true condition, Memphis at last listened to an exposition of Waring's system of sewerage and resolved to give it a trial. After an experiment of three years, the municipal council and the engineers of that city have come to the unanimous conclusion that the method is perfect in every particular, and that the sanitary condition of the city has improved in a wonderful degree since its introduction. The success obtained in Memphis has led other cities to adopt the Waring system. No city in the world has a better plan of sewerage than the great French metropolis. Who has not heard of her subterranean life? The traveler who, stopping in Paris, neglects to visit the "Egouts," misses one of the wonders of the age: a network of tunnels, wherein aqueducts, gas pipes, telephone and telegraph wires, steamers and tramways, sewer carts, etc., are, as it were, in perpetual motion. Notwithstanding these wonderful improvements challenge the admiration of men, many imperfections have been discovered in their structure as well as in their modus agendi. It has been found that the sewers proper have, in several portions of the city, very slight inclinations. They are small and badly ventilated Water pipes and telegraph wires occupy the upper part of the canal, and, at the lower part, infected material accumulates. The municipal council of Paris, in its session of the 11th of April, 1884, decided to remedy the existing evil by the adoption of Waring's system. "With this method," says Mr. Pontzen, "all the excremental matters and household liquids will be discharged *into the sewer*, wherever the condition of the sewers is suitable; they will be sent *through the sewer*, that is to say, by special conduits located wherever possible in the interior of the large sewers, where their immediate delivery into the sewer itself would not be admissible, these special conduits to deliver *into* the sewer as soon as a point is reached where the necessary conditions for the rapid and complete removal of the discharge of such affluents is assured.

"This is one of the great advantages of Waring's system of sewerage, that it can as well be established in isolated sections, constituting an auxiliary and an economical complement of the great system of sewers suited to receive fresh fœcal matter and household waste, as it can, by itself alone, be extended for the complete drainage of whole quarters or of entire cities.

"Whatever may be the extension of a series of sewers according to Waring's system, it retains always, by reason of its exclusion of storm water, the great advantage of requiring only small diameters and reasonable inclinations, in which the volume of flow undergoes only slight variations, and for the cleansing of which relatively small quantities of water suffice.

"The establishment and maintenance of a system of sewers according to Waring's system has, therefore, in all cases, the advantage of being economical."

This action of the municipal council of Paris is but the addition of another laurel leaf to the wreath that encircles the brow of American genius and industry, and we congratulate Mr. Waring upon the great success he has achieved. This action is also a lesson and an example which it were well for other cities to reflect upon and, in many instances, to imitate. Notwithstanding her national pride was somewhat involved; notwithstanding millions had been spent in the construction of a system which it was supposed would meet all the requirements of public sanitation, yet proved in many ways imperfect, the city of Paris did not hesitate to acknowledge her errors and to call to her assistance the brains and pluck of an humble American citizen! Salus populi, suprema lex esto! said she. Far from weakening under mortification, she boldly undertook to rectify the errors of previous construction regardless of cost!

A great duty is now more than ever incumbent upon every city, town and hamlet of America! Cholera will, sooner or later, land on our shore. We, of the South, cannot be ready too soon to meet the grim destroyer. Our old plantation doctors say negroes are his preferred food! What is the sanitary condition of the Southern cities? If we are to judge of it by what taxes our senses in the Gate City of the South, it would look as though every preparation had been made to secure his headquarters therein and make his sojourn no less fruitful than durable.

*Sabbath Bells*—For Sunday schools, prayer, praise and gospel meetings, by Wm. B. Blake, with A. J. Showalter, B. F. Nysewwandet and Chas. E. Prior as special contributors. Published by Fireside Friend Publishing Co., Springfield, Ohio.

An excellent book of songs, containing beautiful selections, adapted to superintendents and choristers, and gems ot song which lovers of music cannot fail to appreciate.

*Memoir of the Nature of Diphtheria*, by Drs. H. C. Wood and H. F. Formad, Philadelphia. Appendix A: Report of the National Board of Health for 1882.

This is an interesting and instructive illustrated pamphlet, treating of, 1st, the structure of the diphtheritic membrane; 2d, on the clinical relation of micrococci to diphtheria; 3d, natural history of micrococci; 4th, relations of the micrococci to diphtheria; 5th, on the relations of diphtheria and its micrococcus to other diseases.

The points for and against the bacterian theory of diphtheria are strongly presented, and leave us still in doubt upon the question. With all that has been said of the germ theory of disease, a large proportion of the profession reject it, claiming that the germs are accompaniments, merely, of diseased states, and not the causes of the disease. They hold the same relation and subserve the same purpose as scavengers in the microscopic world as do the maggot and other larvæ in the visible world.

